# Stratification of a medium secure forensic care pathway according to risk and need: A study from dundrum hospital

**DOI:** 10.1192/j.eurpsy.2021.1002

**Published:** 2021-08-13

**Authors:** N. Basrak, Y. Khogali, R. Twomey, C. O’Leary, D. Prashant, M. Elhassan Elamin, H. Kennedy, M. Davoren

**Affiliations:** 1 National Forensic Mental Health Service, Central Mental Hospital Dundrum, Dundrum, Ireland; 2 The Dundrum Centre For Forensic Excellence, Trinity College Dublin, Dublin, Ireland

**Keywords:** Risks and needs, Stratification

## Abstract

**Introduction:**

Secure forensic mental health services have a dual role, to treat mental illness and reduce violent recidivism. Those admitted to secure forensic services have a significant history of violence and treatment needs in multiple domains including psychiatric illness, violence and other areas such as substance misuse and physical health.

**Objectives:**

The aim of this study was to ascertain if the units in a medium secure forensic hospital are stratified according to individual risks and needs. We also aimed to clarify if there were differences in the symptom level, risks and needs of those with and without community leave and to clarify the risks and needs of the female patients and ID patients.

**Methods:**

This is a cross sectional study a cohort of patients (n=138) in a secure forensic hospital.

**Results:**

There was a total of 138 patients, the majority of whom were male (n=123, 89.1%). The most common diagnosis was schizophrenia (n=99, 71.7%). Placements in the care pathway of the medium secure forensic hospital were associated with level of symptomatology (PANSS positive), dynamic violence risk (F=26.880,P<0.001), DUNDRUM-3 therapeutic programme completion (F=44.067,P<0.001), and DUNDRUM 4 recovery (F=59.629,P<0.001). Patients with community leave had better scores than those without leave on violence risk (F=77.099, P<0.001), therapeutic programme completion (F=116.072, P<0.001) and recovery (F=172.211, P<0.001).

**Conclusions:**

Stratifying secure forensic psychiatric hospitals according to individual risks and needs provides in-patient care in the least restrictive setting appropriate for individuals, however niche groups such as female forensic patients and ID patients may need special consideration.

